# Compound K Induces Endoplasmic Reticulum Stress and Apoptosis in Human Liver Cancer Cells by Regulating STAT3

**DOI:** 10.3390/molecules23061482

**Published:** 2018-06-19

**Authors:** Xuan Zhang, Silin Zhang, Qitong Sun, Wenjun Jiao, Yan Yan, Xuewu Zhang

**Affiliations:** College of Medicine, Yanbian University, Yanji 133000, China; zxfly1028@163.com (X.Z.); zhangchuchu1103@163.com (S.Z.); single0124@163.com (Q.S.); jwj0237@163.com (W.J.); 18243392601@163.com (Y.Y.)

**Keywords:** ginsenoside, CK, apoptosis, STAT3, ERS, hepatoma

## Abstract

The ginsenoside compound K (20-*O*-β-d-glucopyranosyl-20(*S*)-protopanaxadiol; CK) is an intestinal bacterial metabolite of ginseng protopanaxadiol saponin that has been reported to induce apoptosis in many cancer cells; however, the precise mechanisms of its activity in human hepatocellular carcinoma (HCC) cells remain unclear. Herein, we demonstrated that CK inhibited the growth and colony formation of HepG2 and SMMC-7721 cells, phenotypes that were mediated by inducing apoptosis. Meanwhile, CK showed lower toxicity in normal hepatoma cells. After treating HepG2 and SMMC-7721 cells with CK, p-STAT3 levels decreased, the three branches of the unfolded protein response were activated, and levels of endoplasmic reticulum stress (ERS)-related proteins were increased. We also revealed that CK decreased the DNA-binding capacity of STAT3. Moreover, silencing STAT3 with CRISPR/Cas9 technology enhanced CK-induced ERS and apoptosis. Finally, we showed that CK inhibited the growth of liver cancer xenografts with little toxicity. Mice bearing human HCC xenografts that were treated with CK showed increased GRP78 expression and decreased p-STAT3 levels. Taken together, these data showed that CK induced ERS and apoptosis by inhibiting p-STAT3 in human liver cancer cells; thus, CK might be a potential therapeutic candidate for human HCC.

## 1. Introduction

Ginseng is a valuable medicinal herb in Asian countries that has been used for centuries as a panacea to enhance stamina and promote longevity [1,2]. Ginsenosides, or compounds extracted from ginseng, are the major components of ginseng and exhibit useful pharmacological activities including anti-inflammatory, antitumor, anti-dementia and anti-allergic effects [3,4,5]. Among the ginsenosides, compound K (20-*O*-β-d-glucopyranosyl-20(*S*)-protopanaxadiol; CK) is a protopanaxadiol metabolite derived from the deglycosylation of the ginsenosides Rb1, Rb2 and Rc by intestinal bacteria [6,7]. Several studies have shown that CK can attenuate hepatic lipid accumulation [8], produce hepatoprotective effects [9], and induce apoptosis in various cancer cells [10].

The signal transducers and activators of transcription (STAT) family includes STAT1, 2, 3, 4, 5a, 5b and 6, and is closely related to cell growth and differentiation [11]. STAT3 was observed to be hyperactive in cancer where it promotes tumorigenesis by enhancing cell proliferation and angiogenesis in various malignant tumors including liver, lung, and breast cancers [12,13,14]. When phosphorylated (activated), STAT3 translocates into the nucleus and binds specific DNA sequences to promote target gene transcription. The downstream targets of STAT3 include many gene products, such as vascular endothelial growth factor and proteins involved in cell survival and apoptosis. Numerous studies have shown that inhibiting STAT3 activation may have anticancer effects [15,16,17].

The endoplasmic reticulum (ER) plays essential roles in calcium homeostasis, protein folding and protein translocation [18]. Various conditions compromise ER homeostasis and induce the aggregation of unfolded or misfolded proteins, which provokes endoplasmic reticulum stress (ERS) and the unfolded protein response (UPR) to degraded misfolded proteins, promote the correct folding of proteins, and re-establish ER homeostasis [19,20,21]. However, when ERS is severe or prolonged, UPR promotes ERS-associated signaling cascades that stimulate apoptosis [22]. The UPR is mediated by the ER chaperone (glucose-regulated protein 78, GRP78) and three ER-transmembrane stress sensors: protein kinase-like ER kinase (PERK), activating transcription factor 6 (ATF6) and inositol-requiring enzyme 1 (IRE1), all of which bind to GRP78. When ER stress occurs, GRP78 dissociates from the three sensors and initiates the UPR [23]. Moreover, in many cell types, the UPR is also associated with ER-associated protein caspase-4 activation [24]. A previous study showed that caspase 4 activation initiated a caspase cascade that induced apoptosis [25].

Both STAT3 inhibition and ERS activation are involved in natural product-induced apoptosis. However, little is known about the relative importance of STAT3 and ERS for this apoptotic cascade. Herein, we investigated the anti-HCC effects of CK, it mechanisms of action, and the regulation of STAT3 and ERS activation during this process.

## 2. Results

### 2.1. CK Inhibited the Growth of HepG2 and SMMC-7721 Cells by Inducing Apoptosis

**Figure 1:**
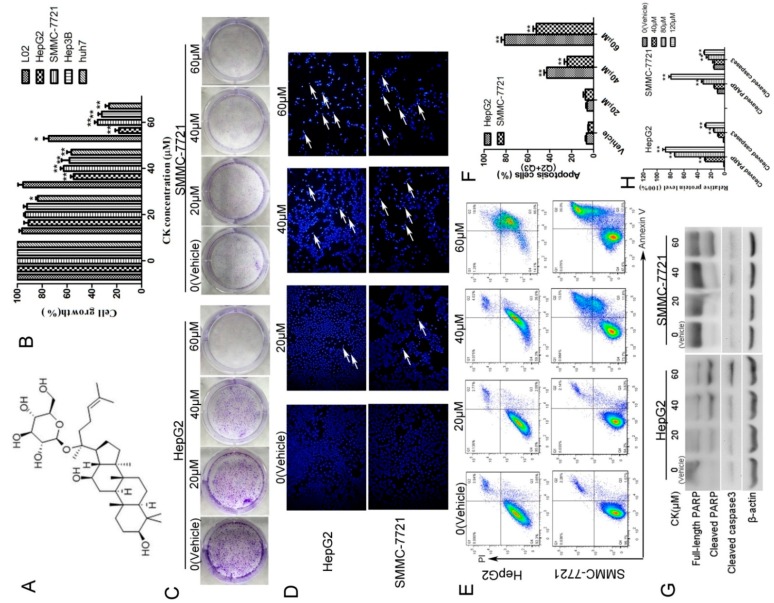
CK inhibited the growth of HepG2 and SMMC-7721 cells by inducing apoptosis. (**A**) Chemical structure of CK. (**B**) HepG2, SMMC-7721, Hep3B, Huh7 and L02 cells were treated with 0, 20, 40, and 60 μM CK for 48 h, and cell viability was measured by the MTT assay; Statistics were done by one-way ANOVA and Dunnett text. * *p* < 0.05, ** *p* < 0.01 vs. Vehicle. (**C**) HepG2 and SMMC-7721 cells were treated with 0, 20, 40, and 60 μM CK for 48 h, and then the cells were cultured in fresh medium for another 7 d. Colonies were stained with crystal violet and imaged. (**D**) HepG2 and SMMC-7721 cells were stained with Hoechst and observed by fluorescence microscopy (200× magnification). (**E**) Apoptotic cells were assessed by flow cytometry following Annexin V/PI staining. (**F**) Quantitative analysis of the data from (**E**) to give the percent of apoptosis cells by flow cytometry. Results are shown as mean ± SD; Statistics were done by one-way ANOVA and Dunnett text. * *p* < 0.05, ** *p* < 0.01 vs. Vehicle. (**G**) HepG2 and SMMC-7721 cells were treated with CK (0, 20, 40, and 60 μΜ) for 48 h, and cleaved caspase 3 and PARP were detected by western blotting. (**H**) Quantification of the data in (**G**). Cleaved caspase 3 and cleaved PARP levels were determined relative to β-actin. Data are presented as mean ± SD; Statistics were done by one-way ANOVA and Dunnett text. * *p* < 0.05, ** *p* < 0.01 vs. Vehicle.

To examine the effects of CK on the growth of human HCC cells, we treated four human liver cancer cell lines (HepG2, SMMC-7721, Hep3B and Huh7) and one normal liver cell line (L02) with CK at concentrations ranging from 20–60 μM for 48 h and MTT (3-(4,5-dimethyl-2-thiazolyl)-2,5-diphenyl-2-*H*-tetrazolium bromide) assay was used. The results showed that CK effectively decreased the growth of liver cancer cells in a dose-dependent manner (Figure 1B). Meanwhile, CK at 20 and 40 μM did not significantly decrease the growth of L02 cells compared with the vehicle-only group. The IC50 values of CK for HepG2, SMMC-7721, Hep3B, and Huh7 cells were 40.45, 48.36, 45.55 and 41.93 μM, respectively. Colony-forming assays showed similar results; CK treatment inhibited the growth of colonies (Figure 1C). Hoechst staining indicated that cell death-associated bright blue/white nuclear fluorescence was increased with increasing CK doses (Figure 1D). Annexin V/propidium iodide (PI) double staining was used to quantify the apoptosis of HepG2 and SMMC-7721 cells. The results showed that CK significantly increased apoptosis in a dose-dependent manner in both cells lines (Figure 1E,F). Meanwhile, levels of the apoptosis-associated proteins cleaved PARP and cleaved caspase3 were markedly increased following CK treatment (Figure 1G,H).

### 2.2. CK Downregulated p-STAT3 Levels in Different HCC Cell Lines

**Figure 2:**
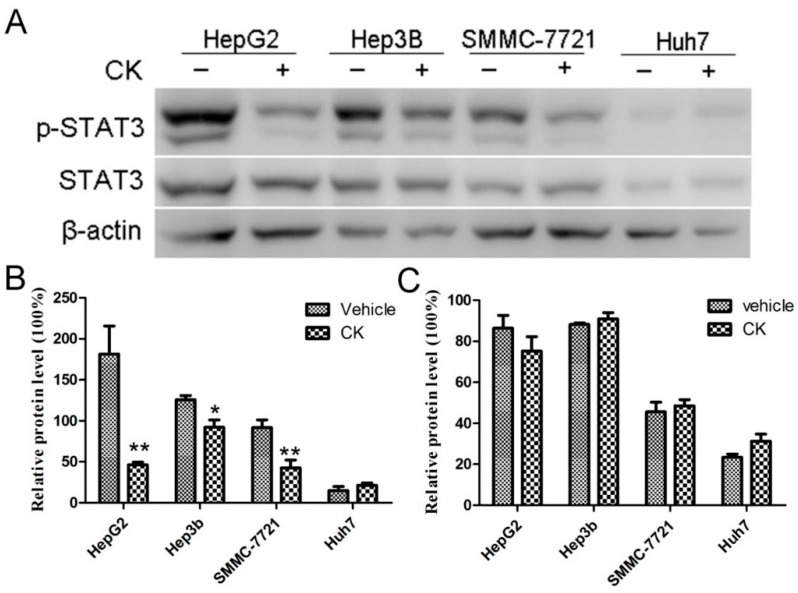
CK reduced p-STAT3 levels in different HCC cell lines. (**A**) Western blot analysis of STAT3 and p-STAT3 levels in different HCC cell lines. (**B**,**C**) Quantification of (B) p-STAT3 and (**C**) STAT3 levels normalized to β-actin. Data are presented as mean ± SD; Statistics were done by one-way ANOVA and Dunnett text. * *p* < 0.05, ** *p* < 0.01 vs. Vehicle.

STAT3 overactivation is known to contribute to tumor development by increasing cancer cell proliferation, survival, angiogenesis, and metastasis. Phosphorylation of tyrosine 705 (Tyr705) is related to the oncogenic status of STAT3. A previous study has showed that the HCC tissue exhibited a higher nuclear staining of p-STAT3 (tyr705) than the adjacent non-tumorous hepatocytes in IHC assay [26]. Thus, we examined the impact of CK on p-STAT3 and STAT3levels in the different HCC cell lines (HepG2, Hep3B, SMMC-7721 and Huh7). As shown in Figure 2A, p-STAT3 levels were partially decreased in various HCC cell lines following treatment with 40 μM CK at 48 h. CK most significantly decreased p-STAT3 levels in HepG2 (*p* < 0.01), Hep3B (*p* < 0.05) and SMMC-7721cells (*p* < 0.01) (Figure 2B), so we used HepG2 and SMMC-7721cells for further experiments.

### 2.3. CK Inhibited p-STAT3 Expression in HepG2 and SMMC-7721 Cells

**Figure 3:**
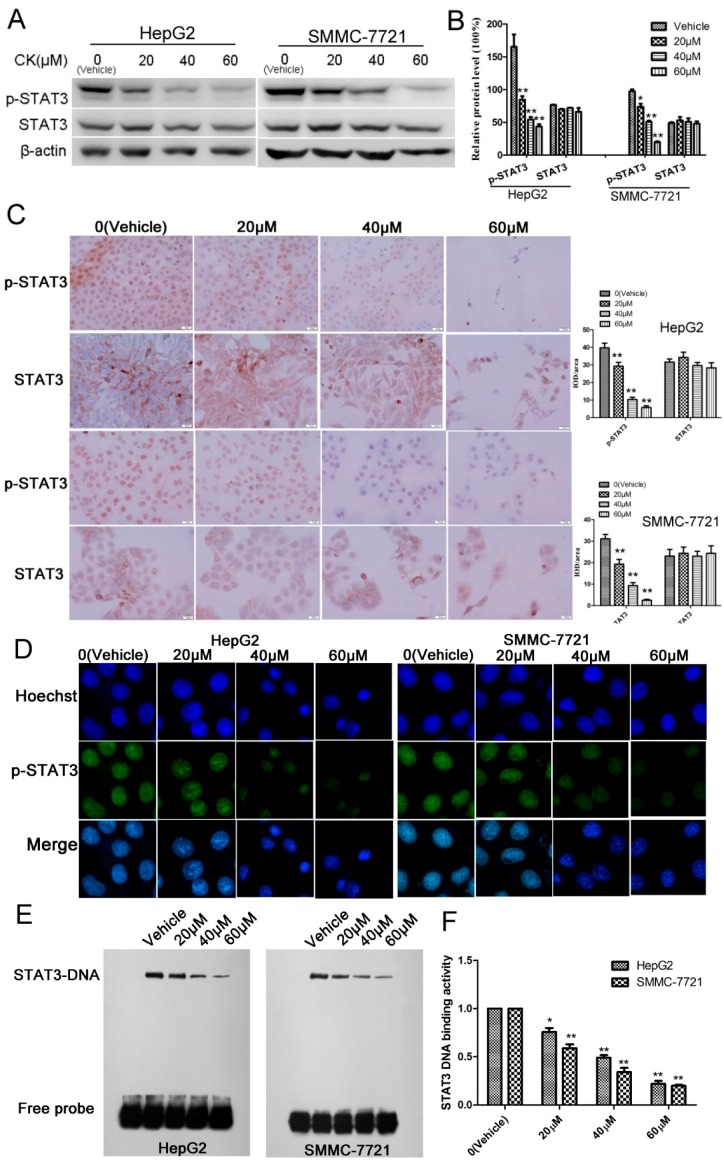
CK inhibited p-STAT3 activity in HepG2 and SMMC-7721 cells. (**A**) HepG2 and SMMC-7721 cells were treated with CK (0, 20, 40, and 60 µΜ) for 48 h, and STAT3 and p-STAT3 levels were detected by western blot. (**B**) Quantification of the western blot data in (**A**) relative to β-actin. Data are presented as mean ± SD; Statistics were done by one-way ANOVA and Dunnett text. * *p* < 0.05, ** *p* < 0.01 vs. Vehicle. (**C**) Immunocytochemistry of STAT3 and p-STAT3 in HepG2 and SMMC-7721 cells after CK treatment (400× magnification). Data analysis is calculated by ImagePro-Plus software. Statistics were done by one-way ANOVA and Dunnett text. * *p* < 0.05, ** *p* < 0.01 vs. Vehicle. (**D**) Immunofluorescence was performed to further clarify p-STAT3 localization. (**E**) EMSA to determine the STAT3 DNA-binding activity after CK treatment in HepG2 and SMMC-7721 cells. (**F**) Quantification of the EMSA results in (**E**) using Image Quant software (Amersham). Data are presented as mean ± SD of three measurements; Statistics were done by one-way ANOVA and Dunnett text. * *p* < 0.05, ** *p* < 0.01 vs. Vehicle.

To investigate the levels and sub-cellular localization of STAT3 and p-STAT3, HepG2 and SMMC-7721 cells were treated with 0, 20, 40, and 60 μM CK for 48 h. The results showed that p-STAT3 levels were significantly reduced in a dose-dependent manner in response to CK treatment (Figure 3A,B). Immunocytochemistry (ICC) and immunofluorescence (IF) clearly indicated that STAT3 was localized in the cytosol and that p-STAT3 was localized in the nucleus (Figure 3C,D). Furthermore, to examine the DNA binding activity of STAT3, electrophoretic mobility shift assays (EMSAs) were performed. EMSA showed that CK inhibited STAT3 DNA-binding activity in a dose-dependent manner in HepG2 and SMMC-7721cells (Figure 3E,F).

### 2.4. CK Induced ERS in HepG2 and SMMC-7721 Cells

**Figure 4:**
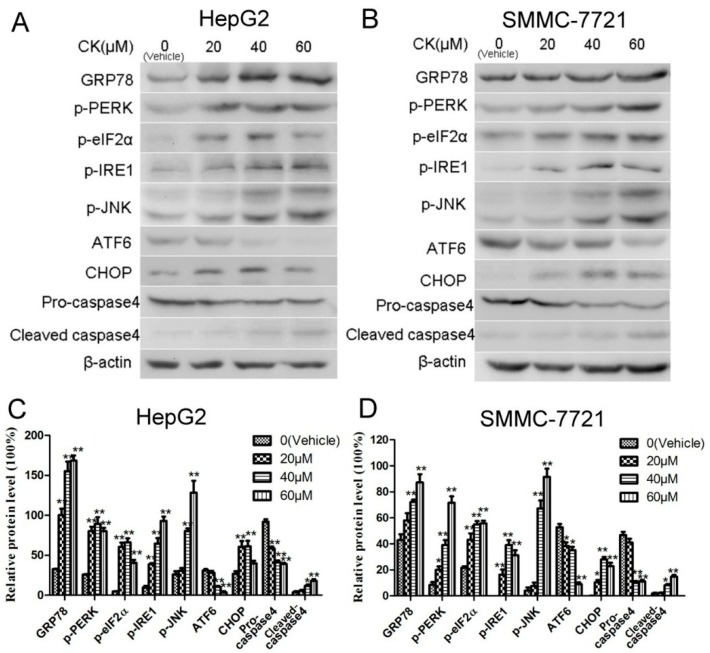
CK induced ERS and evoked UPR in HepG2 and SMMC-7721 cells. (**A**,**B**) Western blot analysis of GRP78, p-PERK, p-eIF2α, p-IRE1, p-JNK ATF6, CHOP, and caspase 4. (**C**,**D**) Quantification of western blot results in (**A**,**B**) normalized to β-actin. Data are presented as mean ± SD; Statistics were done by one-way ANOVA and Dunnett text. * *p* < 0.05, ** *p* < 0.01 vs. Vehicle.

Previous studies have reported that ERS plays an important role in the apoptosis induced by Saponin compounds [27]. In our study, the expression of GRP78 and CHOP (signature ERS markers) were upregulated following CK treatment in HepG2 and SMMC-7721 cells. Additionally, the three UPR signaling pathways were also active. As shown in Figure 4A,B, CK significantly increased levels of phosphorylated (p)-PERK (Thr980), p-eIF2α (Ser51), p-IRE1 (S724) and p-JNK (Thr183/Tyr185), illustrating activation of the PERK and IRE1 pathways. Furthermore, ATF6 levels were diminished, as it presumably translocated to the Golgi where it was cleaved (Figure 4A,B). Meanwhile, levels of pro-caspase4 were markedly decreased, while cleaved caspase4 levels increased with CK treatment (Figure 4C,D). Collectively, these results revealed that CK induced ERS in HepG2 and SMMC-7721 cells.

### 2.5. STAT3 Inhibition Enhanced ERS and Apoptosis in HepG2 and SMMC-7721 Cells

**Figure 5:**
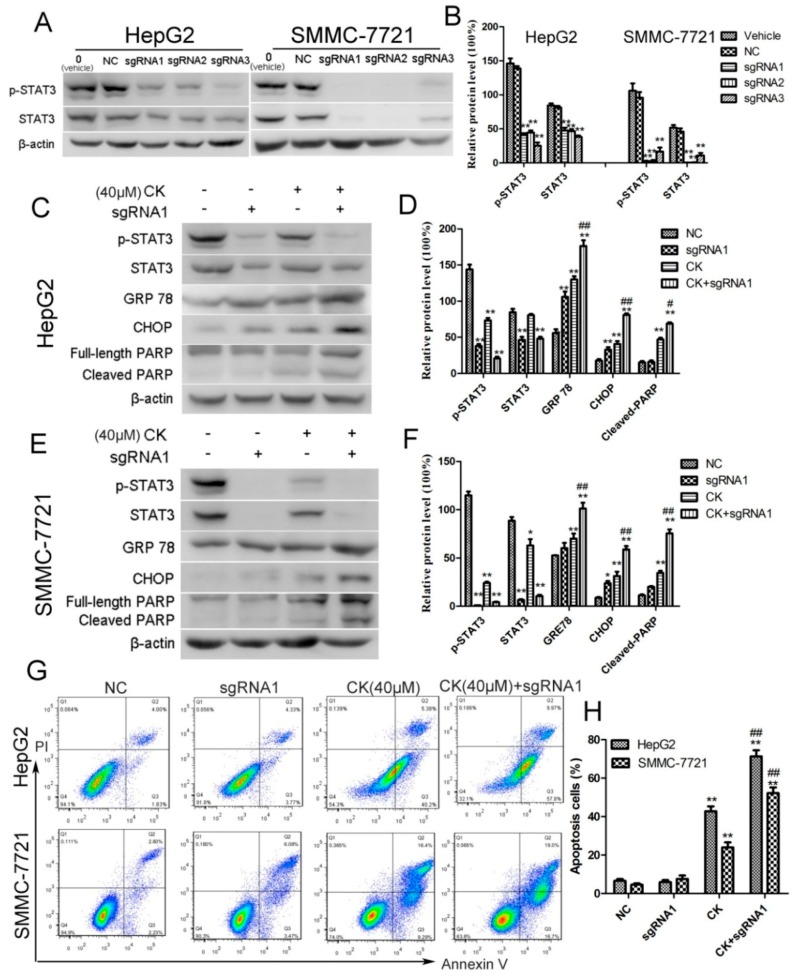
Silencing STAT3 enhanced ERS and apoptosis in HepG2 and SMMC-7721 cells. (**A**) STAT3 was knocked out using CRISPR/Cas9 technology. Western blot analysis for p-STAT3 and STAT3. (**B**) Quantification of the western blot data from (**A**) normalized to β-actin. Data are presented as mean ± SD; Statistics were done by one-way ANOVA and Dunnett text. * *p* < 0.05, ** *p* < 0.01 vs. Vehicle. (**C**) HepG2 or STAT3-sgRNA1-transfected cells were treated with CK. Western blot analysis for p-STAT3, STAT3, GRP78, CHOP, and PARP. (**D**) Quantification of the western blot data in (**C**) normalized to β-actin. Data are presented as mean ± SD; Statistics were done by one-way ANOVA and Dunnett text. * *p* < 0.05, ** *p* < 0.01 vs. NC; ^#^ *p* < 0.05, ^##^ *p* < 0.01 vs. CK. (**E**) SMMC-7721 or STAT3-sgRNA1-transfected cells were treated with CK. Western blot analysis for p-STAT3, STAT3, GRP78, CHOP, and PARP. (**F**) Quantification of the western blot data in (**E**) normalized to β-actin. Data are presented as mean ± SD; Statistics were done by one-way ANOVA and Dunnett text. * *p* < 0.05, ** *p* < 0.01 vs. NC; ^#^ *p* < 0.05, ^##^ *p* < 0.01 vs. CK. (**G**) Apoptotic cells were assessed by flow cytometry following Annexin V/PI staining. (H) Quantitative analysis of the percent of apoptosis cells using the flow cytometry data from (**G**). Results are shown as mean ± SD; Statistics were done by one-way ANOVA and Dunnett text. * *p* < 0.05, ** *p* < 0.01 vs. NC; ^#^ *p* < 0.05, ^##^ *p* < 0.01 vs. CK.

To investigate whether inhibiting STAT3 mediated ERS and apoptosis following CK treatment, we knocked out STAT3 using CRISPR/Cas9 technology. Three small guide RNAs (sgRNAs) were designed against targets within STAT3 and their efficiencies were tested in HepG2 and SMMC-7721 cells. As shown in Figure 5A,B, STAT3 and p-STAT3 levels were inhibited by all three sgRNAs. We chose sgRNA1 (5′-CTGCTGCTTCTCCGTCACCA-3′) to conduct the further experiments. The results showed that silencing STAT3 significantly upregulated the ERS signature proteins GRP78 and CHOP in response to CK (Figure 5C–F). Meanwhile, cleaved PARP levels also increased with CK treatment in STAT3-sgRNA1-transfected HepG2 and SMMC-7721 cells (Figure 5C–F). Furthermore, flow cytometry results showed that inhibiting p-STAT3 significantly increased the proportion of apoptotic cells in STAT3-sgRNA1-transfected cells following CK treatment comparing to CK group. (Figure 5G,H). Taken together, blocking STAT3 activation enhanced ERS and apoptosis in CK-treated HepG2 and SMMC-7721 cells.

### 2.6. CK Inhibited In Vivo Tumor Growth, and STAT3 and ERS Were Involved in the Antitumor Effects

**Figure 6:**
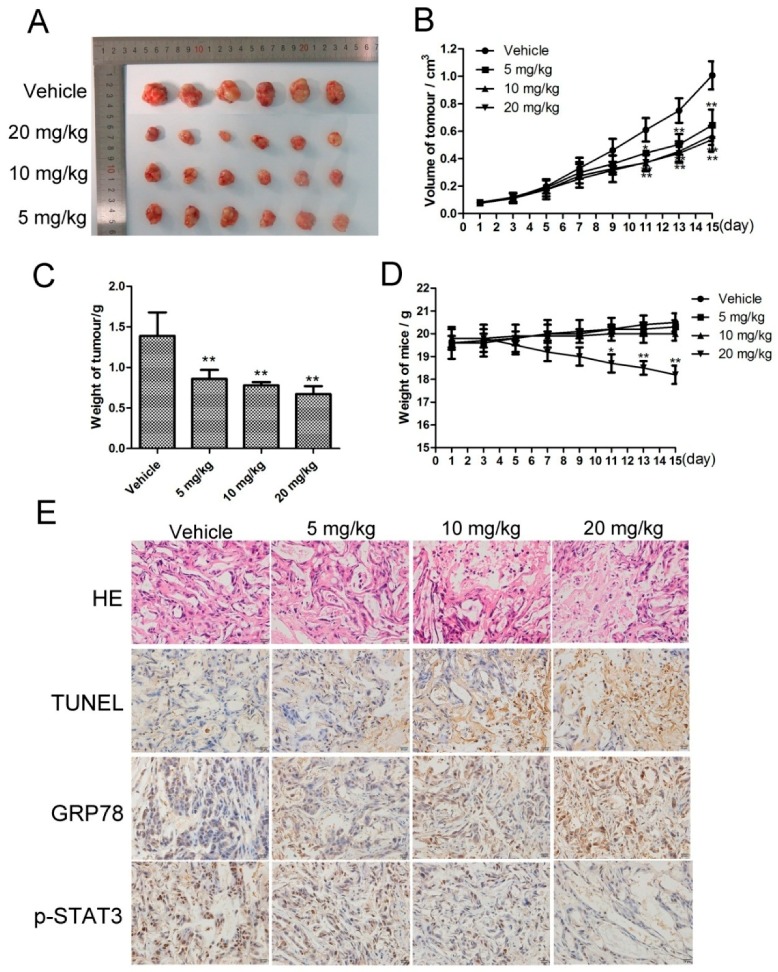
CK inhibited in vivo liver cancer xenograft tumor growth. (**A**) At the end of the experiment, resected tumors from each group were collected and imaged. (**B**) Tumor volumes were calculated after treatment with CK or normal saline (Vehicle) once every two days for 15 d. Data are presented as mean ± SD; Statistics were done by one-way ANOVA, Dunnett text and Bonferroni text. * *p* < 0.05, ** *p* < 0.01 vs. Vehicle. (**C**) Tumors weights for each group were measured on the last day of the experiment (day 15). Data are presented as mean ± SD; Statistics were done by one-way ANOVA and Dunnett text. * *p* < 0.05, ** *p* < 0.01 vs. Vehicle. (**D**) Body weights of each group were measured once every two days for 15 d. Data are presented as mean ± SD; Statistics were done by one-way ANOVA and Dunnett text. * *p* < 0.05, ** *p* < 0.01 vs. Vehicle. (**E**) Tumor samples were subjected to HE staining, TUNEL assays and IHC analysis for GRP78 and p-STAT3 at the end of the experiment.

Based on the pharmacological effects of CK in vitro, we injected SMMC-7721 cells into nude mice to investigate the in vivo antitumor activity of CK. When tumors had reached approximately 50–100 mm^3^, the mice were treated with normal saline (vehicle) or 5, 10, and 20 mg/kg CK. The results showed that CK inhibited tumor growth in mice; the tumor inhibition rates were 38.64%, 44.01% and 51.79% for 5, 10 and 20 mg/kg CK, respectively (Figure 6A). Compared with vehicle controls, tumor weights and volumes were significantly reduced following CK treatment (Figure 6B,C). However, there are not significant differences of tumor volumes between the CK treatment groups. Additionally, there were no significant differences in body weight among the vehicle and 5 and 10 mg/kg CK-treated groups (Figure 6D). Furthermore, hematoxylin and eosin (HE) staining and TUNEL (TdT-mediated dUTP Nick-End Labeling) assays showed that necrotic and apoptotic cells in the tumor tissues were significantly increased following CK treatment compared with the vehicle group (Figure 6E). Meanwhile, immunohistochemical (IHC) staining showed that CK treatment upregulated GRP78 expression and downregulated p-STAT3 levels (Figure 6E). Taken together, CK inhibited tumor growth and induced apoptosis in vivo, activities that might be related to regulating STAT3 and ERS in HCC cells.

## 3. Discussion

HCC is the fifth most commonly diagnosed cancer and the second leading cause of cancer-related mortality worldwide [28,29]. Because of late diagnosis and the limited number of effective drugs, HCC has a poor prognosis. Meanwhile, systemic chemotherapy is still the mainstay of HCC treatment. Thus, there is an urgent need to develop more effective and less toxic chemotherapeutic drugs or targeted treatment approaches. The ginsenoside CK has been shown to exhibit significant anticancer activity in various cancer cell lines, including HCC cell lines [30,31]; however, the precise mechanisms of CK activity remain unclear human hepatoma cells.

In this study, we demonstrated that CK decreased the growth of liver cancer cells in a dose-dependent manner. Study has showed CK exhibits a low toxicity in normal hepatoma cells [30]. Similarly, CK showed lower toxicity in a normal human liver cell line in our study. Additionally, CK induced apoptosis in HepG2 and SMMC-7721 cells, as determined by morphological changes, Annexin V/PI staining, and the expression of apoptotic proteins. Moreover, CK-treated mice showed a significant tumor growth inhibition ratio. TUNEL assays indicated a higher percentage of apoptotic cells in the tumor tissues of CK-treated mice. Collectively, CK exhibited effective antitumor effects in vitro and in vivo, suggesting it might be a potential candidate drug for HCC therapy.

STAT3 is a transcription factor that plays critical roles in HCC [32]. Several studies have shown that blocking STAT3 sensitizes cancer cells to apoptotic stimuli [33,34]. To explore the effect of CK on STAT3 in HCC, we detected STAT3 and p-STAT3 (Tyr705) levels in several HCC cell lines following CK treatment. These results showed that p-STAT3 (Tyr705) levels were decreased in four HCC cell lines. To mechanistically explore the effects of CK on STAT3, we chose HepG2 and SMMC-7721 cells for subsequent cell-based experiments. These data showed a remarkable reduction in STAT3 activation in response to CK treatment in HCC cell lines as well as in liver tumor xenograft models. Together, these data showed that CK suppressed the expression of p-STAT3 in HCC cells. Furthermore, CK inhibited STAT3 DNA-binding activity to regulated STAT3 target genes that are involved in tumor survival and apoptosis.

ERS is a new pathway that is recently shown to regulate apoptosis and the cytotoxicity of natural products to cancer cells [35]. ERS-induced apoptosis is partially mediated by upregulating CHOP [36]. When ERS occurs, activated PERK and IRE1 phosphorylate their downstream targets eIF2α and JNK, respectively, which induce CHOP expression [37]. Additionally, ATF6 is activated by proteolytic cleavage inside the Golgi apparatus, and cleaved ATF6 fragments translocate to the nucleus where they bind X-box-binding protein 1 (XBP1), to directly activate CHOP transcription [38]. Moreover, Caspase-4 localizes to the ER and is primarily activated during ERS-induced apoptosis [39]. In this study, CK increased GRP78 and CHOP expression; meanwhile, we also observed activation of all three UPR branches (PERK, IRE1a, and ATF6) and increasing levels of cleaved caspase4 in HepG2 and SMMC-7721 cells. Thus, CK-induced apoptosis was closely associated with ERS.

Several studies have shown that inhibiting p-STAT3 increased ERS-associated apoptosis [40,41]. In this study, we found that CK-induced ERS in liver cancer cells was associated with reduced p-STAT3. The importance of STAT3 in mediating CK-induced ERS in HepG2 and SMMC-7721 cells was further demonstrated by knocking out STAT3. We found that levels of the ERS protein markers GRP78 and CHOP were increased, and that CK-induced apoptosis was increased when STAT3 was removed via CRISPR/Cas9 technology. These data demonstrated that inhibiting p-STAT3 by CK can affect ERS and apoptosis in HepG2 and SMMC-7721 cells. However, further studies are needed to confirm the precise mechanisms between STAT3 and ERS.

**Figure 7:**
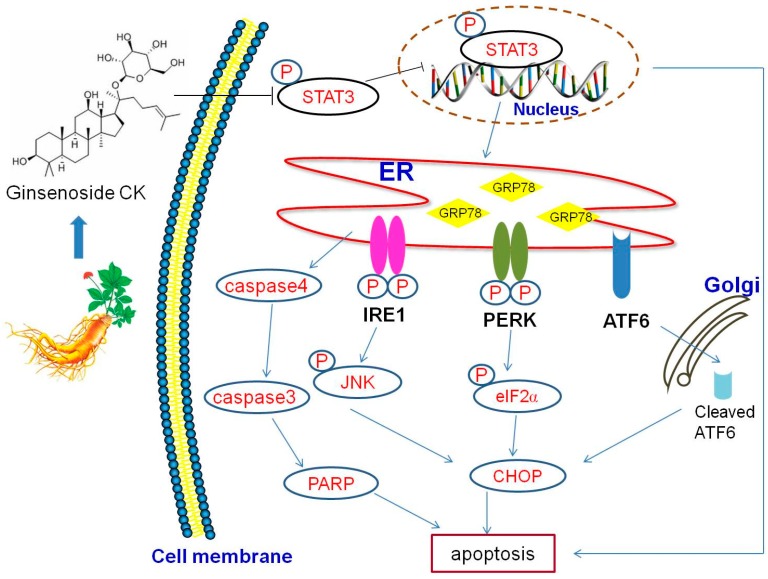
Schematic illustration of the potential pathway underlying the anticancer activity of CK in human HCC cells.

In conclusion, our study revealed the significant antitumor activity of CK and its potential underlying mechanisms of action. We demonstrated the anti-tumor mechanisms of CK on affecting ERS and apoptosis in this process as modulated by STAT3 (Figure 7). These results indicated that CK might be a potential therapeutic candidate for HCC and that the mechanisms of its anticancer activity were regulating STAT3 activation and the ERS pathway.

## 4. Materials and Methods

### 4.1. Reagents

Compound K (Figure 1A) (purity ≥ 98%) was purchased from Shanghai yuanye Bio-Technology Co., Ltd. (Shanghai, China). Dulbecco’s modified Eagle’s medium (DMEM) and penicillin-streptomycin were purchased from Gibco-BRL (Grand Island, NY, USA), and fetal bovine serum (FBS) was obtained from BI. Rabbit-polyclonal antibodies of STAT3, p-STAT3 (Tyr705), GRP78, p-IRE1(S724), Cleaved-caspase3 were purchased from Abcam (Cambridge, MA, USA). Rabbit-polyclonal antibodies of p-PERK (Thr980), p-eIF2α (Ser51), p-JNK (Thr183/Tyr185), ATF6, CHOP and β-actin were purchased from Cell Signaling Technology (Danvers, MA, USA). The secondary antibody was purchased from ZSGB-Bio Co., Ltd. (Beijing, China). The chemicals used were 3-(4,5-dimethylthiazol-2-yl)-2,5-diphenyltetrazolium bromide (MTT) (Sigma, St. Louis, MO, USA), Hoechst 33258 (Beyotime, Jiangsu, China), Annexin V-FITC/propidium iodide (PI) apoptosis Detection Kit (Beyotime, China).

### 4.2. Cell Culture and Growth Assay

Human hepatoma cell lines (HepG2, SMMC-7721, Hep3B, Huh7) were purchased from Key GEN Co., Ltd. (Nanjing, China) and cultured in DMEM with 10% FBS, 100 U/mL penicillin-streptomycin at 37 °C in a humidified (5% CO_2_, 95% air) incubator. Cells were plated in 96-well plates (5 × 10^3^/100 µL/well) and subjected to CK (0, 20, 40, 60 µM). MTT (5.0 mg/mL, 20 µL) was added after the cells were treated with CK for 48 h, then 150 µL DMSO was added into each well. The optical density (OD) was measured at 490 nm respectively. The cell viability value was calculated using the following formula: OD sample/OD blank × 100%.

### 4.3. Colony-Forming Assay

Cells were seeded into 6-well plates at a density of 500 cells per well. After treatment with CK (0, 20, 40, 60 µM) for 48 h, the cells were replaced with fresh medium and cultured for another 7 days. Colonies were fixed with 4% paraformaldehyde (Sigma, Ronkonkoma, NY, USA), stained with 0.5% crystal violet (Solarbio, Beijing, China) for 15 min. Colonies were visualized and photographed.

### 4.4. Fluorescent Staining

HepG2 and SMMC-7721 cells grown in 6-well plates were treated with CK for 48 h, incubated with Hoechst 33258 for 25 min and washed with phosphate-buffered saline (PBS). Fluorescence images were captured by fluorescence microscope (Olympus, Tokyo, Japan).

### 4.5. Annexin V/PI Double Staining

Cells were seeded into 6-well plates (3 × 10^5^ cells/well), and then treated with CK (0, 20, 40, 60 μM) for 48 h. The cells were harvested, washed twice with cold PBS, and resuspended in 195 μL of binding buffer. Then, 5 μL annexin V-FITC and 10 μL PI were added, and the mixture was incubated for 15 min in the dark. After added 300 μL PBS, the samples were detected by flow cytometer. The results were analyzed with the BD FACSCalibur™ system (BD Biosciences, Franklin Lakes, NJ, USA).

### 4.6. ICC Staining Assay

Cells were seeded in 6-well plates (1 × 10^5^ cells/well) and cultured overnight. They were treated with CK (0, 20, 40, 60 μM) for 48h. Briefly, HepG2 and SMMC-7721 cells were blocked in 3% hydrogen peroxide for 15 min. They were incubated with primary antibodies of STAT3 (1:100), p-STAT3 (Tyr705) (1:250) overnight at 4 °C. After washing with PBS, the cells were incubated with secondary antibodies for 1 h at 37 °C. Then the cells were stained with 3, 3′-diaminobenzidine and counter-stained with hematoxylin. The results were photographed by a light microscope (Olympus, Tokyo, Japan) at 400× magnification. The results were characterized quantitatively via digital image analysis using ImagePro-Plus (Media Cybernetics, Silver Spring, MD, USA). IOD/area = Integrated optic density (IOD)/positive areas [42].

### 4.7. IHC Staining Assay

After the mice were sacrificed, tumors were excised, embedded in paraffin, sectioned, and rehydrated. After antigen retrieval, the sections were incubated in 3% hydrogen peroxide for 10 min. Then, the sections were blocked with goat serum for 30 min and incubated with primary antibody p-STAT3 (1:250) and GRP78 (1:100) overnight at 4 °C. The next day, the sections were incubated with secondary antibodies for 1 h at 37 °C. Then they were stained with 3, 3′-diaminobenzidine and counter-stained with hematoxylin. The results were photographed by a light microscope (Olympus, Japan) at 400× magnification.

### 4.8. IF Assay

Cells were treated the indicated amounts of CK for 48 h and collected for standard immunofluorescence assays using a confocal laser scanning microscope (Olympus, Japan) at 600× magnification.

### 4.9. EMSA

Nuclear protein and cytoplasm protein was extracted from the CK-treated cells. The proteins were incubated with the DNA probe. The DNA-protein complex was separated on a 6% pre-chilled non-denaturing polyacrylamide gel at 4 °C. Then the mixtures were transferred onto a positively charged membrane and cross-linked. The gel shifts were visualized with a gel imaging system (BIO-RAD, Hercules, CA, USA).

### 4.10. CRISPR/Cas9 Mediated STAT3 Knockdown

CRISPR-Cas9 small guide RNA lentiviral vector targeting human STAT3 were purchased from Shanghai Genechem Co., Ltd. (Shanghai, China). HepG2 and SMMC-7721 cells were seeded into 6-well plate (3 × 10^4^ cells/well) in 2 mL media and cultured overnight. The cells were treated with 25 μL lentiviral vector (4 × 10^8^ TU/mL) in 1 mL media with 5 μg/mL Ploybrene. 24 h later, cells were continuously cultured for another 24 h after changing conventional culture medium. Then, 6 μg/mL Puromycin were used to screening transfected cells. The expression of STAT3 and p-STAT3 was confirmed by western blot.

### 4.11. Western Blot Analysis

After treatment HepG2 and SMMC-7721 cells with various concentrations of CK, the total protein was obtained by RIPA lysis buffer (Solarbio, Beijing, China). The proteins were separated by sodium dodecyl sulfate-polyacrylamide gel electrophoresis (SDS-PAGE) (100 V, 120 min) and transferred to a polyvinylidene fluoride (PVDF) membrane (100 V, 30–90 min). Subsequently, The membranes were blocked with skimmed milk (5%) and then incubated overnight at 4 °C with the following antibodies: STAT3 (1:1000), p-STAT3 (1:5000), GRP78 (1:1000), CHOP (1:500), p-PERK (1:1000), p-eIF2α (1:1000), p-IRE1 (1:1000), p-JNK(1:1000), ATF6(1:500), cleaved-caspase 3 (1:500), PARP(1:500) and β-actin(1:1000). The next day, the membranes were incubated with anti-rabbit secondary antibody (1:5000) for 2 h at room temperature. At last, after ECL incubated, the target proteins were tested using BIO-RAD imaging system (BIO-RAD, Hercules, CA, USA).

### 4.12. Animals and Tumor Implantation

BALB/c female nude mice (30 days, 18–22 g) were purchased from Shanghai Lingchang Biological Technology Co., Ltd., (Shanghai, China). The tumor implantation experiments were authorized by the Institutional Animal Care and Use Committee of Yanbian University. Animals were housed in laminar flow room maintained a constant temperature. SMMC-7721 cells (1 × 10^7^ cells/mL) were injected subcutaneously into the right side of axillary of mice. The length and width were measured by caliper of each tumor for estimate relative tumor volume (RTV) as follows: RTV = 1/2 (length × width^2^). When tumors reached a volume of 50–100 mm^3^, the mice were randomized to groups (*n* = 6) that intravenously received CK (5, 10, or 20 mg/kg) or normal saline (Vehicle) every day for 15 days. Then the animals were sacrificed, and the tumors were removed and weighed for the studies of histology and proteins expression. Tumor growth inhibition ratio (%) = (TW_Vehicle_ − TW _treatment_)/TW_Vehicle_ × 100%. (TW_Vehicle_ and TW_treatment_ were the average of tumor weights of the Vehicle and CK treatment groups, respectively.).

### 4.13. HE Staining

The paraffin sections of xenograft tumor tissues with or without CK treatment were stained with hematoxylin and eosin (H&E). Each image of the sections was captured using a light microscope (Olympus, Japan) at 400× magnification.

### 4.14. TUNEL Assay

TUNEL assay were performed with the paraffin sections of xenograft tumor tissues with or without CK treatment according to the instructions of the manufacturer (Key GEN, Nanjing, China).

### 4.15. Statistical Analysis

All experiments were repeated three times. The data were expressed as the mean ± SD, and differences between groups were analyzed by one-way analysis of variance (ANOVA) and Student’s *t*-test. The results were considered to be statistically significant when the *p*-value was less than 0.05. Statistical Package for SPSS 19.0 and Graph Pad Prism 5.0 software was used to analyze the results.
